# RNA Interference Analysis of *Legionella* in *Drosophila* Cells: Exploitation of Early Secretory Apparatus Dynamics

**DOI:** 10.1371/journal.ppat.0020034

**Published:** 2006-04-28

**Authors:** Marion S Dorer, Donald Kirton, Joel S Bader, Ralph R Isberg

**Affiliations:** 1 Department of Molecular Biology and Microbiology, Tufts University, Boston, Massachusetts, United States of America; 2 School of Medicine, Tufts University, Boston, Massachusetts, United States of America; 3 Department of Biomedical Engineering, Johns Hopkins University, Baltimore, Maryland, United States of America; 4 Howard Hughes Medical Institute, Tufts University, Boston, Massachusetts, United States of America; University of California Berkeley, United States of America

## Abstract

Legionella pneumophila translocates multiple bacterial effector proteins into host cells to direct formation of a replication vacuole for the bacterium. The emerging consensus is that formation of this compartment involves recruitment of membrane material that traffics between the endoplasmic reticulum (ER) and Golgi. To investigate this model, a targeted approach was used to knock down expression of proteins involved in membrane trafficking, using RNA interference in *Drosophila* cells. Surprisingly, few single knockdowns of ER–Golgi transport proteins decreased L. pneumophila replication. By analyzing double-stranded RNAs in pairs, combinations were identified that together caused defects in intracellular replication, consistent with the model that membrane traffic funnels into the replication vacuole from multiple sources. In particular, simultaneous depletion of the intermediate compartment and Golgi-tethering factor transport protein particle together with the ER SNARE protein Sec22 reduced replication efficiency, indicating that introduction of lesions at distinct sites in the secretory system reduces replication efficiency. In contrast to knockdowns in secretory traffic, which required multiple simultaneous hits, knockdown of single cytosolic components of ER-associated degradation, including Cdc48/p97 and associated cofactors, was sufficient to inhibit intracellular replication. The requirement for the Cdc48/p97 complex was conserved in mammalian cells, in which replication vacuoles showed intense recruitment of ubiquitinated proteins, the preferred substrates of Cdc48/p97. This complex promoted dislocation of both ubiquitinated proteins and bacterial effectors from the replication vacuole, consistent with the model that maintenance of high-level replication requires surveillance of the vacuole surface. This work demonstrates that L. pneumophila has the ability to gain access to multiple sites in the secretory system and provides the first evidence for a role of the Cdc48/p97 complex in promoting intracellular replication of pathogens and maintenance of replication vacuoles.

## Synopsis

Legionella pneumophila is a pathogenic bacterium that causes Legionnaires pneumonia. Immune cells, called macrophages, engulf the bacterium and attempt to kill it. *Legionella* avoids this killing and instead grows inside the macrophage, creating a growth niche using host cell components. The bacterium directs the formation of its replication niche by injecting bacterial proteins, called effectors, into the host cell. These effectors hijack host functions. In this study, the authors identify some of the host pathways that the bacterium hijacks. The authors used macrophage-like cells derived from fruit flies because protein function can be disrupted in these cells using a technique called RNA interference, which destroys the RNA messages that encode for proteins, resulting in directed loss of these proteins. Candidate proteins were chosen to disrupt based upon previous knowledge about the biology of *Legionella*. This report highlights two observations that contribute to our understanding of the biology of *Legionella.* Surprisingly, the absence of some host components could be tolerated because other host components could take their place. One exception to this rule was a protein complex on the outside of the *Legionella* replication vacuole that may help the bacterium deliver its proteins to appropriate sites in the host cell.

## Introduction

The agent of Legionnaires pneumonia, the Gram negative *Legionella pneumophila,* creates a replication vacuole that is dependent on the function of the Dot/Icm protein translocation machine, a member of the specialized type IV conjugative secretion machinery family [1,2]. This system transports at least 30 different protein substrates across target membranes [3–7]. Dot/Icm function results in recruitment of secretory vesicles, mitochondria, and endoplasmic reticulum (ER) that form the L. pneumophila–containing vacuole (LCV) [8,9]. Consistent with translocated proteins mediating membrane recruitment for these compartments, two translocated proteins, LidA and RalF, target transport between the ER and Golgi [3–5,8–10]. Deletions of individual translocated substrates cause minor defects in intracellular replication, indicating that the bacterium may use functionally redundant proteins to exploit multiple host pathways for intracellular replication [3–6].

Little is known about the host factors that contribute to the formation of the LCV, although there is accumulating evidence indicating host proteins regulating traffic between the ER and Golgi play an important role in intracellular replication. Formation of the LCV appears to require vesicle budding from the ER, as overproduction of mutants misregulated in the function of the small GTPase Sar1 interfere with both budding and L. pneumophila intracellular replication [9]. Dominant negative inhibitors of Arf1 and Rab1 activity, which regulate multiple steps in ER and Golgi membrane traffic, also interfere with formation of the LCV [8,9,11]. In addition, RalF, one of the translocated substrates of Dot/Icm, is a guanine nucleotide exchange factor for Arf1. Finally, secretory pathway components Sec22b, Rab1 [8,11], Rab5c [12], and Arf1 [3] all localize to the LCV, suggesting these components may play a role in formation of this compartment. Adding to the complexity of the LCV is that it shows some characteristics of an autophagic compartment [13,14]. As autophagy may use proteins normally involved in promoting early secretory traffic, it may be difficult to distinguish the replication vacuole from a modified form of an autophagic compartment [15].

Host cells can control the fate of proteins translocated by bacterial pathogens by promoting ubiquitin-dependent proteolysis. Ubiquitination of the Salmonella typhimurium protein SopE results in its destruction by the proteasome, downregulating actin ruffling events promoted by this translocated protein [16]. In addition, the occasional S. typhimurium breaks out of its replication vacuole and is polyubiquitinated, allowing proteasome-dependent restriction of cytosolic replication [17]. Although not previously implicated in controlling replication of intracellular pathogens, one host protein that recognizes ubiquitinated substrates is the Cdc48/p97 AAA ATPase [18]. Cdc48/p97 forms at least two separate complexes [19,20]. A complex of Cdc48/p97, Ufd1, and Npl4 has chaperone-like activity for diverse processes, including ER-associated degradation (ERAD) [19,21]. A second complex of Cdc48/p97 and p47 regulates fusion of mitotic Golgi [22] and transitional ER membranes [23]. Given the close connection between the ER and LCV formation, this complex may have access to proteins that control L. pneumophila replication.

*Drosophila* tissue culture cells have emerged as a model system for identifying host proteins that contribute to replication of intracellular pathogens [24–28] because they are amenable to interference with double-stranded RNA (dsRNA) [29]. In addition, unlike other RNA interference systems, *Drosophila* cells allow the analysis of knockdowns of multiple genes simultaneously by adding dsRNA against more than one target in a single culture [30]. This allows both the analysis of functional redundancy and the generation of bypass suppression. In this report, we created a library of dsRNAs that targeted the secretory pathway, endocytic functions, and ER-related processes, allowing identification of factors that support L. pneumophila intracellular replication. In addition, we introduced multiple dsRNAs into a single *Drosophila* cell culture to identify combinations of knockdowns that interfered with intracellular replication. This approach allowed the identification of multiple host factors that control L. pneumophila replication.

## Results

### Simultaneous Interference of Multiple Steps in the Secretory Pathway Depresses L. pneumophila Replication

As L. pneumophila replicates in target cells as diverse as amoeba and human macrophages [31], we used dsRNA treatment of *Drosophila* tissue culture cells to identify host proteins that modulate intracellular replication [24,26,27]. L. pneumophila replicated to high levels in *Drosophila* Kc167 cells and replication depended on the Dot/Icm type IV secretion machinery *(dotA),* indicating that replication in these cells faithfully mimicked L. pneumophila interactions with other host cells. (Figure S1) [32]. To analyze replication in *Drosophila* cells, a thematic approach was pursued, using a selected subset of 73 dsRNAs that were targeted against genes encoding proteins associated with the early secretory pathway, ER dynamics, and endocytic transport (Table 1), because L. pneumophila traffics to an ER-bound compartment and intercepts membrane from ER exit sites [8,9]. After *Drosophila* Kc167 cells were treated with dsRNA for 3 d, the cells were incubated with L. pneumophila–green fluorescent protein (GFP) for 48 h, fixed, and screened visually for increased or decreased intracellular replication based on quantification of vacuole size.

**Table 1:**
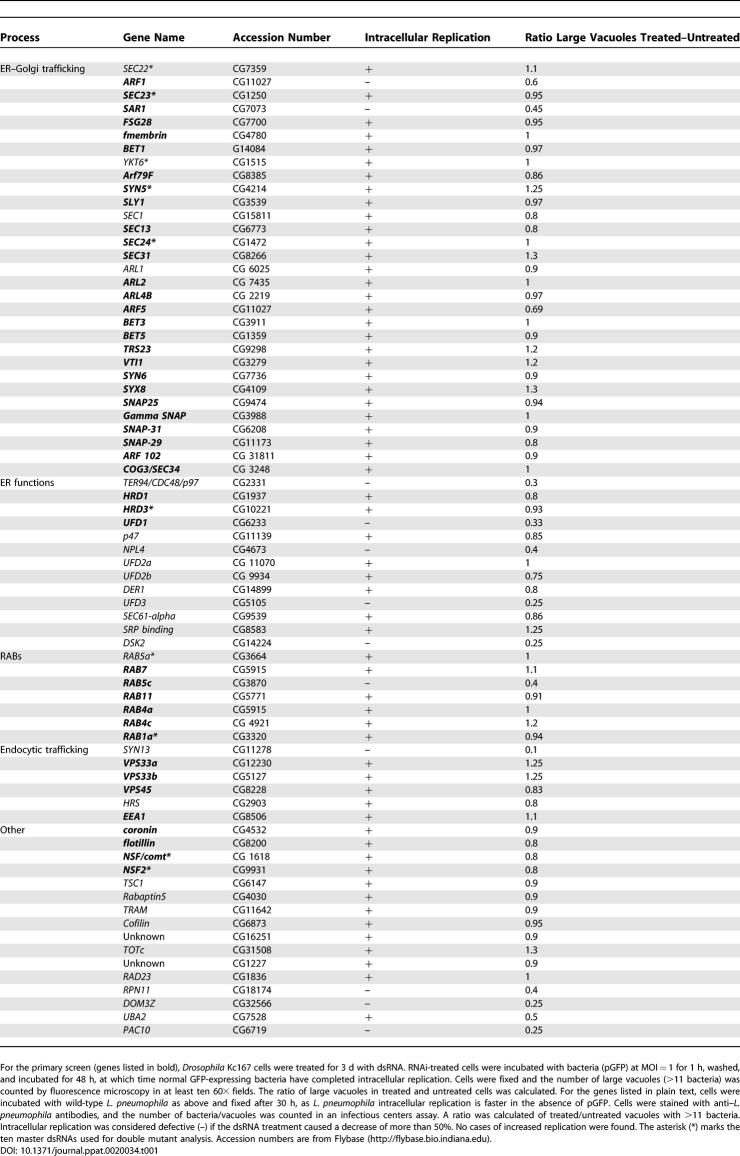
dsRNA Treatments Tested for Their Effect on L. pneumophila Replication

Few dsRNAs targeting ER–Golgi transport depressed bacterial replication. Only dsRNA against the small GTPase Sar1 and Arf1 depressed intracellular replication (Table 1), consistent with the observation that dominant-negative Sar1 and Arf1 decreased L. pneumophila replication in mammalian cells [9]. However, there still appeared to be significant replication even in cells treated with these dsRNAs. That other ER–Golgi transport targets did not effect intracellular replication was surprising, as we found that Golgi morphology was disrupted by treatment of several of the dsRNAs directed against proteins involved in export of vesicles from the ER (unpublished data). This indicates that much lower concentrations of these proteins are necessary to support L. pneumophila replication than are required to maintain normal organelle biogenesis.

Since only a small number of single dsRNAs targeting ER–Golgi transport decreased L. pneumophila intracellular replication, it seemed possible that multiple pathways may deliver membrane to the LCV, so that interfering with only one pathway may not be enough to cause replication defects. This model was tested by simultaneously treating *Drosophila* cultures with two dsRNAs that target different aspects of the secretory pathway. Ten master dsRNAs (Table 1), most of which were involved in budding from the ER or movement of ER-derived vesicles into downstream compartments, were each combined with the 73 dsRNA targeting secretory components to find those combinations that decreased L. pneumophila replication. After allowing for 24 h of replication of L. pneumophila in the presence of the 730 dsRNA combinations, images of fluorescein-labeled bacteria and DAPI-labeled nuclei were collected, and the fluorescein images were inspected visually for decreased intracellular replication. We found that dsRNAs against members of the transport protein particle (TRAPP) complex consistently enhanced defects in intracellular replication caused by the master dsRNAs without decreasing cell number, as indicated by the DAPI images. The multisubunit TRAPP complex, localized to the ER–Golgi intermediate compartment (IC) and the Golgi, has been implicated in vesicle tethering and activation of the small GTPase Rab1 [33,34] indicating that either this complex, or membrane traffic through these compartments, may play a role in intracellular replication.

**Figure 1:**
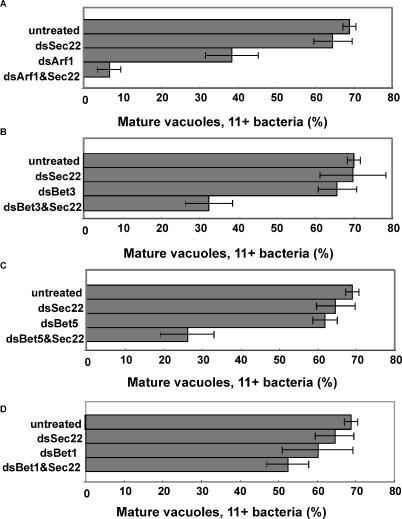
RNAi Targeting Multiple Secretory Components Decreases L. pneumophila Replication dsRNA-treated Kc167 cells were incubated for 1 h with L. pneumophila at MOI = 1, washed, and incubated for 30 h prior to microscopic examination of replication vacuoles. The noted dsRNAs were added to *Drosophila* cells, and allowed to incubate with the cells prior to introduction of *L. pneumophila,* as described (see Materials and Methods). Untreated: no dsRNA added. The mean ± standard error is plotted.

To analyze the effects of lowered expression of TRAPP, dsRNAs targeted against several members of the complex were retested in combination with dsRNA directed against the ER-associated snare protein Sec22, which was one of the original ten master dsRNAs. Sec22 had been previously localized to the LCV and there is evidence that this protein may be involved in formation of the replication vacuole [8,11], although single dsRNA directed against Sec22 had little effect on intracellular replication (Figure 1). After allowing for 24 h of replication, the efficiency of formation of large replication vacuoles was determined after treatment with dsRNAs against Sec22 in combination with TRAPP components Bet3, Bet5, and Trs23 [5]. For comparison, dsRNA against both Sec22 and Bet1, another ER SNARE, was analyzed, as were the dsRNA combination Sec22 and Arf1, a protein thought to be important for LCV formation [3]. None of the single dsRNAs, except Arf1, decreased L. pneumophila replication (Figure 1A). The double combination of Sec22 with TRAPP components Bet3, Bet5 (Figure 1B and 1C) and Trs23 (unpublished data) significantly depressed the number of LCVs harboring 11+ bacteria, even though single dsRNAs targeting these factors had no effect. Double combination of dsRNA against Arf1 and Sec22 had profound effects, reducing the number of LCVs far lower than the single dsRNA against Arf, even though dsRNA against Sec22 had little effect. In contrast, the combination of dsRNA against Sec22 and Bet1, which may target the identical steps in secretory traffic, had no effect on L. pneumophila replication (Figure 1D). Therefore, it appears the strongest defects in intracellular replication can be obtained by targeting multiple steps in the secretory pathway.

**Figure 2:**
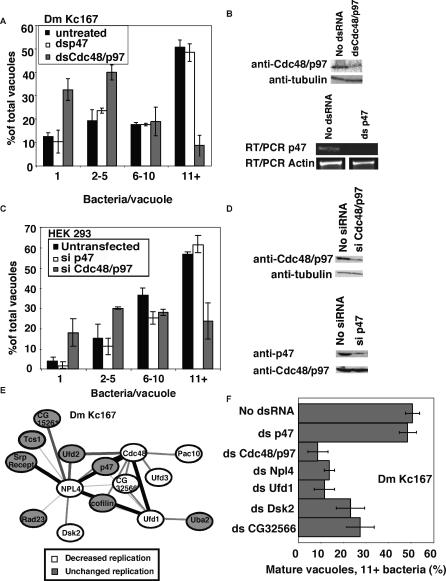
RNAi Targeting Cdc48/p97 and Cofactors Decreases L. pneumophila Replication in *Drosophila* and Human Tissue-Culture Cells (A) dsRNA treated Kc167 cells were incubated for 1 h with L. pneumophila at MOI = 1, washed, and incubated for 30 h prior to microscopic examination of replication vacuoles; mean ± standard error. (B) *Drosophila* Kc167 treated with dsRNA directed against Cdc48/p97, p47 or no dsRNA [30]. Cdc48/p97-treated cells (1 × 10^6^) were lysed in SDS-PAGE buffer, separated by SDS-PAGE electorphoresis, and subjected to Western blotting with anti-Cdc48/p97 [35] and antitubulin (Serotec) as a loading control. RNA from p47-treated cells was collected using trizol reagent (Invitrogen), cDNA was prepared using Superscript reverse transcriptase (Invitrogen) and 25 cycles of PCR were completed against either actin or p47. (C) HEK 293 cells were silenced for p47 or Cdc48/p97 [39], incubated with L. pneumophila at MOI = 2, and washed and incubated for 11 h prior to microscopic examination of replication vacuoles. (D) HEK 293 cells were silenced for Cdc48/p97 or p47 [39] and subjected to Western blotting with anti-p97 or anti-p47. Antitubulin (Serotec) served as a loading control. (E) Network of genes that interact with Cdc48/p97. Lines are color coded and weighted according to predicted confidence score for each interaction [36]. (F) RNAi of Cdc48/p97 cofactors as in (A), except only the mature (11+ bacteria) vacuoles were plotted.

Some single dsRNAs reduced bacterial burden, including those against the endocytic component Syn13, the small GTPase Rab5c, and the multifunctional enzyme Cdc48/p97 (Ter94 in *Drosophila* [35]) (Table 1 **)**. All three phenotypes were retested in an assay similar to Figure 1 (unpublished data and Figure 2A). Although dsRNA directed against Syn13 caused a strong defect in intracellular replication, these cells had markedly lowered viability (unpublished data) and intracellular replication of L. pneumophila was unchanged by dsRNA against Syn13 binding partners Hrs and Eea1 [36] (Table 1), so this was not studied further. Rab5c has been localized to the LCV [12], so it may play a role in L. pneumophila replication, but its function is unknown, unlike that of its closely related isoform Rab5A [37], which had no effect on intracellular L. pneumophila replication.

### Cdc48/p97 Is Required for L. pneumophila Intracellular Replication

The depression in intracellular growth caused by depletion of Cdc48/p97 was studied further, as this represented one of the few examples of single dsRNAs that caused defective intracellular replication of *L. pneumophila,* and no role had been ascribed for this protein in intracellular replication of pathogens. Cdc48/p97 functions in various membrane fusion events and as a chaperone for ERAD [18,19,21]. The normal function of ERAD is to target unfolded proteins to the surface of the ER where they are ubiquitinated, removed by the Cdc48/p97 complex, and destroyed by the proteasome. Cdc48/p97 forms at least two complexes. The first functions in ERAD and includes Npl4 and Ufd1 [18,20]. The second includes p47 and regulates fusion of mitotic Golgi [22,38] and transitional ER membrane [23]. To determine which Cdc48/p97 complex is involved in L. pneumophila replication in *Drosophila* cells, we synthesized dsRNA against p47 and found that it did not effect L. pneumophila replication, whereas dsRNA directed against Cdc48/p97 significantly decreased L. pneumophila replication, as the number of vacuoles harboring 11+ bacteria was depressed and those harboring one bacterium were increased (Figure 2A). Decreased protein level caused by Cdc48/p97 dsRNA treatment was confirmed by Western blot and decreased mRNA level caused by p47 dsRNA treatment was confirmed by RT-PCR (Figure 2B). Therefore, although Cdc48/p97 function is required for high efficiency replication in *Drosophila* cells, formation of a complex between this protein and p47 appears dispensable.

Although the *Drosophila* system has been investigated previously for identification of proteins involved in intracellular replication of mammalian pathogens, there has been no clear demonstration that the identical proteins play any role in supporting intracellular replication in mammalian cells [26,27]. To determine if L. pneumophila requires Cdc48/p97 function in human cells, hairpin small interfering RNA directed against human Cdc48/p97 or p47 [39] was expressed in HEK 293 cells, and intracellular replication was similarly quantified (Figure 2C). Silencing of Cdc48/p97 but not p47 in 293 cells decreased L. pneumophila replication, indicating that Cdc48/p97 function is required for intracellular replication in mammalian cells. Decreased protein level caused by silencing of Cdc48/p97 or p47 was confirmed by Western blot (Figure 2D). Therefore, these results demonstrate that knockdown in *Drosophila* cells predicts defects in L. pneumophila replication observed in mammalian cells.

Silencing of Cdc48/p97 could decrease the efficiency of L. pneumophila replication by decreasing the translocation of effectors or recruitment of ER to the vacuole. Immunostaining of effectors LidA [5] and SidC [4] appeared normal in 293 cells silenced for Cdc48/p97. ER recruitment to LCVs, as measured by immunostaining of LCVs with ER luminal protein calnexin, was equally efficient in 293 cells with silenced p47 or Cdc48/p97 (unpublished data).

### Factors Associated with ERAD Are Required for L. pneumophila Intracellular Replication

Two-hybrid studies of *Drosophila* gene products identified a number of proteins that interact with Cdc48/p97 [36] (Figure 2E). Some are involved in ERAD, and a subset facilitates the chaperone function of Cdc48/p97, whereas others regulate membrane fusion. To further distinguish the Cdc48/p97 membrane fusion and chaperone functions, Kc167 cells were treated with dsRNA directed against partners of Cdc48/p97 (Figure 2E). dsRNA treatment of Npl4, Ufd1, Ufd3, Dsk2, Pac10, and CG32566 significantly decreased L. pneumophila replication (Figure 2E and 2F; Table 1). Ufd1 and Npl4 are cytosolic proteins and have been identified in a large complex with Cdc48/p97 that recognizes protein chains destined for ERAD [18,20], and genetic studies indicate that Dsk2 also functions in ERAD [40]. In contrast, dsRNA directed against membrane channels Der1 and Sec61 alpha [41] had no effect on intracellular replication (Table 1). We found no evidence that the unfolded protein response is activated in L. pneumophila–infected cells, using either a transcriptional reporter or in transcript profiling of infected macrophages (unpublished data). Therefore, this dsRNA genetic analysis indicates that L. pneumophila exploits the modular nature of Cdc48/p97, using only the cytosolic components of ERAD. In addition, Pac10 and CG32566 may be unidentified cofactors of the Cdc48/p97 chaperone, showing that analysis of intracellular replication can potentially identify unknown functions of host proteins.

### Cdc48/p97 Function Is Specific to the LCV

As Cdc48/p97 is required for diverse processes in cells [18,19,21,22], its depletion may simply create a poor host for intracellular replication. To address this question, two intracellular pathogens were tested for their ability to replicate in cells depleted for this protein. Yersinia pseudotuberculosis and Salmonella typhimurium replication were unaffected by dsRNA against Cdc48/p97, Ufd1, Npl4, or p47 in Kc167 cells (Figure S2A and S2B). As a control, we showed that Y. pseudotuberculosis intracellular replication is decreased by dsRNA directed against both *Drosophila* orthologs of the AAA ATPase NSF [42], whereas L. pneumophila replication was unchanged (Figure S2C; NSF, comatose). These observations indicate that this analysis of host pathways distinguished divergent intracellular replication strategies.

**Figure 3:**
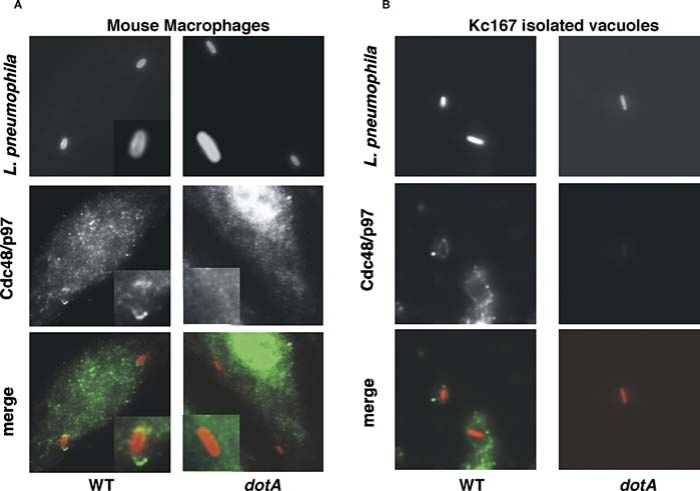
Cdc48/p97 Localizes to the L. pneumophila Vacuole (A) Mouse BMDMs were incubated with L. pneumophila for 1 h at MOI = 1, fixed, and immunostained for human Cdc48/p97 and L. pneumophila. Inset is an enlargement of bacterium in panel. Cdc48/p97 associated with 63% ± 11% vacuoles harboring wild-type bacteria and 1.3% ± 1% *dotA* bacteria. (B) Kc167 cells were incubated with L. pneumophila for 1 h at MOI = 1; replication vacuoles were isolated, fixed, and immunostained with antibodies against *Drosophila* Cdc48/p97 (Ter94) [35] and L. pneumophila. Cdc48/p97 associated with 77% ± 6.9% vacuoles harboring wild-type bacteria and 1.3% ± 0.3% *dotA* bacteria.

Cdc48/p97 associates with the ER [43], so we investigated whether it localizes to the ER-bound LCV in mammalian cells, giving further evidence that the *Drosophila* cell system identifies host components important for L. pneumophila replication within diverse species. Cdc48/p97 associated with 63% ± 11% of vacuoles in mouse bone marrow–derived macrophages (BMDMs). L. pneumophila that lack an intact type IV secretion system *(dotA)* target to the late endosome [2,44] and failed to associate with Cdc48/p97 (1.3% ± 1% vacuoles associated with Cdc48/p97) (Figure 3A). In addition, Cdc48/p97 colocalized with LCVs isolated from Kc167 cells, demonstrating a tight connection of Cdc48/p97 to the replication site (Figure 3B). Therefore, association of this protein with the LCV is evolutionarily conserved from *Drosophila* to mammalian cells.

### Association of Ubiquitinated Proteins with the LCV Is Controlled by Cdc48/p97

**Figure 4:**
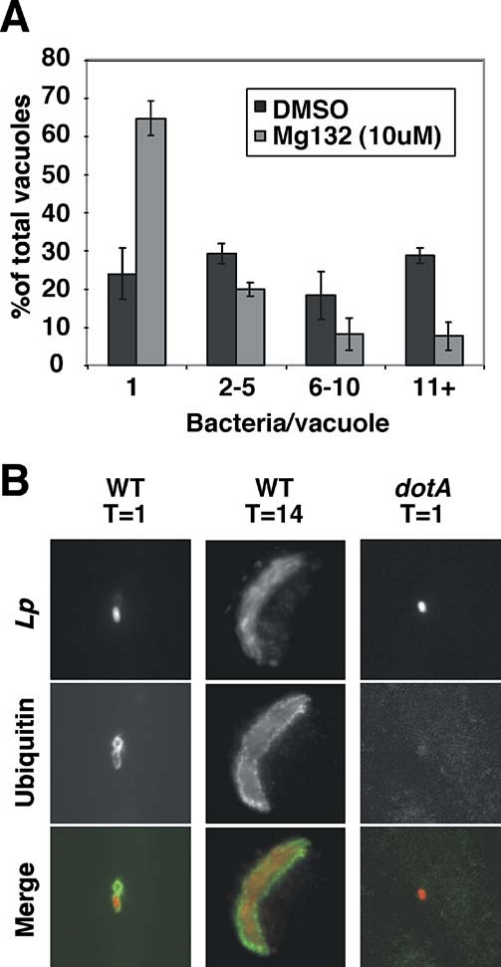
The Proteasome Promotes L. pneumophila Replication and Translocation of Bacterial Effectors causes Ubiquitination of the LCV (A) Mouse BMDMs were pretreated for 1 h with proteasome inhibitor MG-132 or DMSO, incubated with L. pneumophila for 1 h at MOI = 1, washed, and incubated for 14 h. Infectious centers assay was performed as described. (B) Mouse BMDMs were incubated with L. pneumophila for 1 h at MOI = 1, fixed at the indicated time, and immunostained with antipolyubiquitin and anti–*L. pneumophila.*

As Cdc48/p97 delivers incorrectly folded proteins to the proteasome for degradation [19,21], the proteasome may also promote L. pneumophila replication. dsRNA against the proteasome subunit Rpn11 in *Drosophila* Kc167 cells decreased L. pneumophila replication (Table 1). To determine if this requirement was conserved, mouse BMDMs were treated with a proteasome inhibitor (MG-132) for 1 h prior to incubation with bacteria, and quantitative inspection of bacterial yield within individual vacuoles showed that bacterial replication was significantly decreased (Figure 4A). Similar to the knockdown of Cdc48/p97, proteasome inhibition had no effect on avoidance of the endocytic network by the LCV or translocation of the bacterial effector SidC (unpublished data).

The link between L. pneumophila replication and ubiquitin-mediated proteolysis was further investigated because the Cdc48/p97 chaperone binds polyubiquitin, as do cofactors Ufd1 [18], Npl4 [20], and Dsk2 [45]. Mouse BMDMs were infected for 1 h with wild-type L. pneumophila and immunostained with anti-polyubiquitin. Polyubiquitinated proteins associated with 67% ± 8% LCVs in mouse BMDMs containing wild-type bacteria at 1 h after infection and persisted throughout the intracellular replication cycle (T = 14; 79% ± 1.5% LCVs associated with polyubiquitin). In contrast, no polyubiquitinated proteins associated with translocation-defective mutants *(dotA* ^−^ *)* (Figure 4B), showing that translocation of effectors recruits polyubiquitination of the LCV**.** Very little cytosolic polyubiquitin was detected in mouse BMDMs (Figure 4B) in contrast to human U937 or *Drosophila* Kc167 cells, allowing easy detection of a concentrated signal about the LCV. In the other cells, cytosolic polyubiquitin staining obscured the LCV staining, and polyubiquitinated proteins were only detected after staining of LCVs isolated from postnuclear supernatants (unpublished data). The polyubiquitinated proteins may be derived from the host or bacteria; however, the presence of the Dot/Icm system, which translocates bacterial effector proteins, was required.

**Figure 5:**
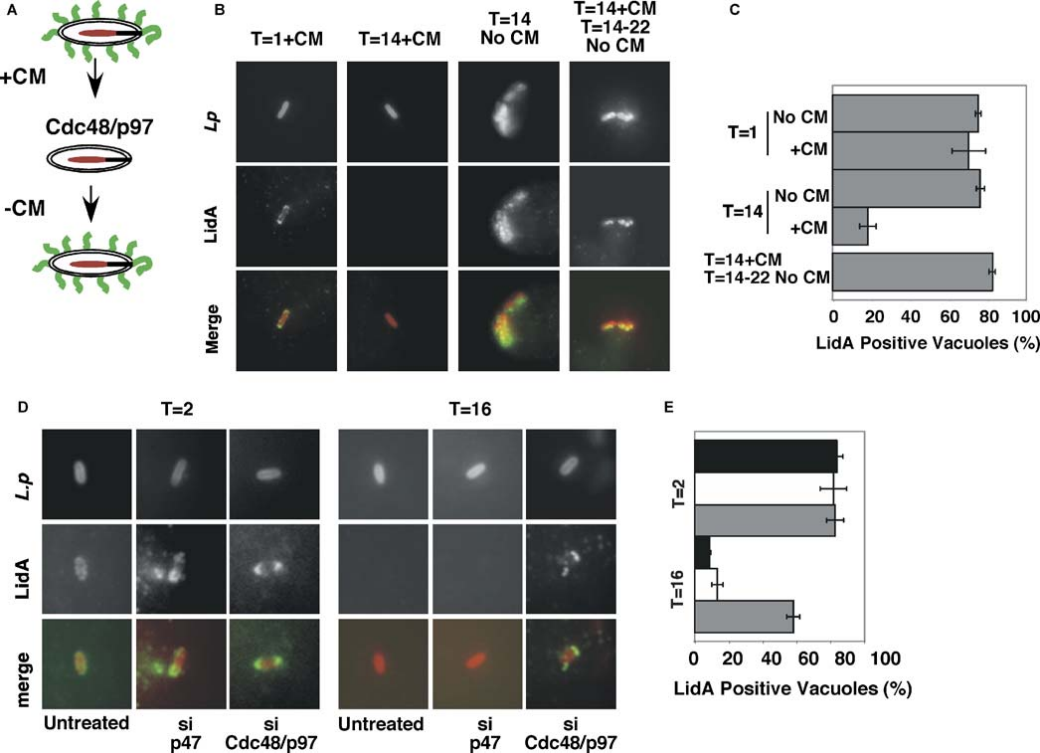
Cdc48/p97 Removes Translocated Effectors from the Replication Vacuole (A) Cdc48/p97 mediates the removal of LidA (green) from the LCV (red) during incubation with CM. (B) Mouse BMDMs were incubated with L. pneumophila at MOI = 1 for 1 h in CM, washed, and fixed, or further incubated in CM as indicated. Cells were immunostained with anti-LidA and anti–*L. pneumophila.* (C) Quantification of LidA-positive vacuoles from (B), showing mean of three replicates ± standard error; at least 100 cells counted per replicate. (D) HEK 293 cells were silenced for Cdc48/p97 or p47 and incubated with wild-type L. pneumophila at MOI = 5 for 2 h in CM, washed, and fixed, or further incubated in CM for 16 h. Intact cells were immunostained for LidA and *L. pneumophila.* (E) Quantification of LidA-positive vacuoles from (D). Untreated (black), si p47 (white), si Cdc48/p97 (gray). Mean of three replicates ± standard error; at least 100 cells counted per replicate.

Although translocation of effectors LidA and SidC appeared normal in cells silenced for Cdc48/p97, its function in ERAD suggested that it might modulate proteins associated with the LCV. Therefore, we assessed whether Cdc48/p97 removed translocated effectors from the LCV. To this end, a protocol for shutting off the translocation of effector LidA was developed, and its removal was followed. LidA is associated with the LCV throughout intracellular replication [5] (see Figure 5B, panel 3). Inhibition of bacterial protein synthesis with chloramphenicol (CM) blocked bacterial replication, but did not disrupt trafficking to the LCV [46] or the initial translocation of LidA (Figure 5A). Treatment with chloramphenicol caused a slow but consistent loss of LidA from the vacuole (Figure 5B). After 1 h of incubation in CM, effector LidA colocalized with the LCV, but disappeared after 14 h of protein synthesis inhibition (Figure 5B and 5C). These observations indicated that effector translocation occurred initially, but continued association of LidA about the LCV required bacterial protein synthesis. After 14 h, CM was removed, and after several hours, the bacteria began replicating and association of LidA with the vacuole was restored (Figure 5B and 5C). Similar results were obtained for polyubiquitin associated with the LCV (unpublished data).

As the Cdc48/p97 chaperone complex removed both ubiquitinated and nonubiquitinated proteins from the ER [18], we investigated whether it removes both polyubiquitinated proteins and translocated effectors associated with the LCV. To determine if Cdc48/p97 removed translocated effector LidA from the LCV we silenced Cdc48/p97 or p47 in 293 cells, incubated the cells with wild-type L. pneumophila in the presence of CM*,* isolated LCVs, and immunostained for LidA. Silencing of Cdc48/p97 in 293 cells stabilized LidA association with the LCV after 16 h of protein synthesis inhibition (Figure 5D and 5E). In contrast, silencing of p47 did not effect LidA association with the LCV (Figure 5D and 5E). Identical results were obtained for the translocated substrate SidC (unpublished data).

**Figure 6:**
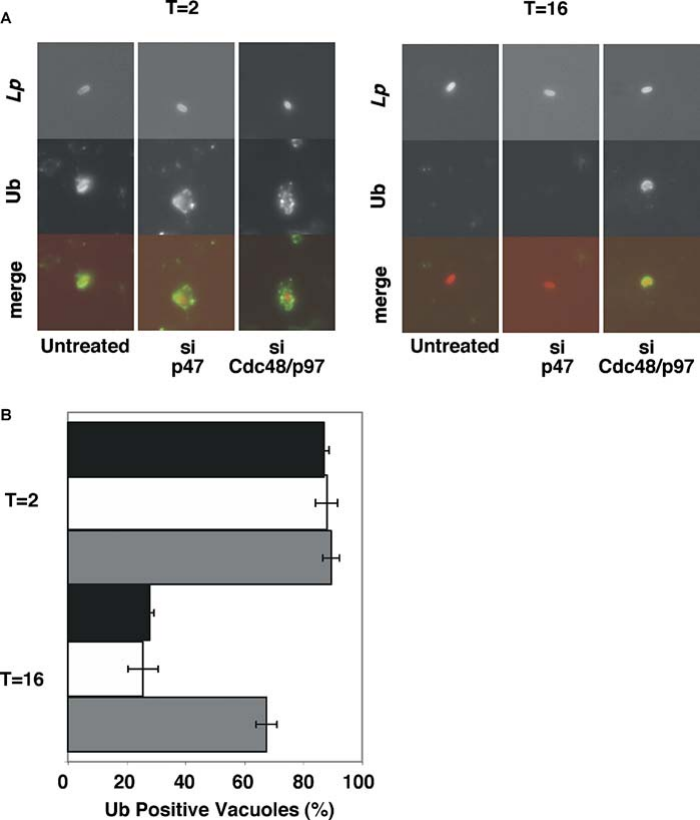
Cdc48/p97 Removes Polyubiquitinated Proteins from the Replication Vacuole (A) HEK 293 cells were silenced for Cdc48/p97 or p47 and incubated with wild-type L. pneumophila at MOI = 5 for 2 hours in CM, washed, and fixed, or further incubated in CM for 16 h. Intact cells were immunostained for polyubiquitinated proteins and *L. pneumophila.* (B) Quantification of polyubiquitin positive vacuoles from (A). Untreated (black), si p47 (white), si Cdc48/p97 (gray). Mean of three replicates ± standard error; at least 100 cells counted per replicate.

We tested whether Cdc48/p97 also removed polyubiquitinated proteins after treatment with protein synthesis inhibitors. After 2 h of CM treatment, polyubiquitin associated with more than 80% of vacuoles isolated from untreated, Cdc48/p97-, or p47-silenced cells (Figure 6A and 6B), but after 16 h, vacuoles associated with polyubiquitin in only 28% (±1.2%) untreated and 25% (±5.1%) silenced p47 cells. In contrast, polyubiquitin associated with more than 67% (±3.6%) of vacuoles isolated from silenced Cdc48/p97 cells (Figure 6A and 6B). Taken together, these observations suggest that Cdc48/p97 participated in the removal of polyubiquitinated proteins and bacterial substrates from the LCV. Our initial experiments have failed to identify ubiquitinated LidA and SidC species (unpublished data).

## Discussion

L. pneumophila exploits host cells for intracellular replication. In the work described here, the high level of functional conservation between *Drosophila* and higher eukaryotes and the unique ability to knock down multiple targets in *Drosophila* cells has revealed unappreciated levels of complexity present in the relationship between the host and this pathogen. At first glance, genome-wide screens would seem to be the most likely to uncover novel host factors required by the bacterium. By choosing a thematic approach, unexpected factors were found to be involved in this process. The possibility of functional redundancy, as implied in our analysis of the ER–Golgi pathway would not have been uncovered in a genome-wide analysis. In addition, we show for the first time that the identical proteins identified as important for replication of a pathogen within *Drosophila* cells are also involved in promoting its replication in mammalian cells. A unifying theme is that L. pneumophila intracellular replication accesses multiple host pathways through a diverse set of effectors encoded by the microorganism, creating a functionally redundant system that is highly resistant to perturbation of either single bacterial or host components. As ERAD processes multiple effector proteins, it may be an Achilles heel for L. pneumophila intracellular replication, whereas the secretory pathway has multiple input sources to the LCV, which can compensate for one another.

Previous work showed that the LCV may capture vesicles that bud from both the ER and the pre-Golgi IC, because dominant inhibitory mutants that potentially affect both anterograde and retrograde traffic interfere with intracellular replication [9]. Two observations are consistent with the idea of that multiple sources of vesicles feed into the replication vacuole. First, few single dsRNAs targeting proteins involved in ER and Golgi traffic disrupted L. pneumophila replication. The rare exceptions were the small GTPases Sar1 and Arf1 (Table 1), perhaps because depletion of these proteins results in defects in multiple steps in membrane trafficking. The second observation is that combinations of dsRNA directed toward genes encoding the TRAPP complex and the ER–Golgi transport protein Sec22 decreased L. pneumophila replication. Alternatively, as residual protein may be present after dsRNA treatment, our observations could be explained as additive effects of a partial decrease in both proteins.

**Figure 7:**
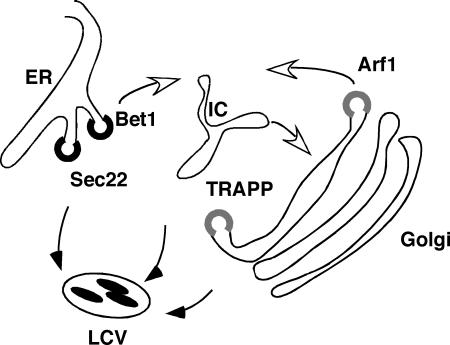
Model for Functional Redundancy The LCV captures secretory vesicles from multiple pathways. Sec22, TRAPP, and Arf1 promote vesicle movement through the IC, and the LCV captures membrane moving from the ER through the IC (white arrows). Alternatively, Sec22 and TRAPP may dock and tether vesicles on the LCV (filled arrows). In either case, *Legionella* replication is decreased when both pathways are disrupted.

In yeast, the TRAPP complex appears multifunctional, facilitating the recognition and tethering of ER-derived vesicles to downstream compartments, as well as promoting activation of the small GTPase Rab1, a regulator of fusion events associated with traffic between the ER and Golgi [34]. There are two possible roles for the TRAPP in L. pneumophila replication. First, similar to its role in membrane trafficking, TRAPP may tether vesicles to the LCV. TRAPP has not been localized in L. pneumophila–infected cells, but such an effect could be mediated through Rab1, which localizes to the LCV, is activated by TRAPP [34], and is required for maximal intracellular replication of L. pneumophila [8,11]. The second possibility is that loss of TRAPP disrupts the function of host cell compartments such as the IC that are required for intracellular replication, preventing the LCV from capturing the membrane it needs to expand from these compartments (Figure 7). Disruption of TRAPP components in yeast causes dilated ER and an accumulation of secretory vesicles [47], suggesting that the ER–Golgi IC does not function properly in its absence. Even so, loss of TRAPP function is not sufficient to abrogate L. pneumophila replication, strongly supporting the idea that multiple redundant pathways create the LCV, and that proteins working upstream of TRAPP in the secretory apparatus play a role in intracellular replication. Use of multiple pathways may help the bacterium insinuate itself into the host secretory pathway without disrupting host secretion, thereby extending the lifetime of the host cell.

That Sec22 and TRAPP synergize to promote intracellular replication is particularly intriguing, as these proteins are resident in different compartments, but both are involved in docking vesicles. There is evidence that Sec22, which localizes on the LCV, is important for L. pneumophila replication in CHO cells, where its function was disrupted by overproduction of a cognate SNARE, membrin [11]. In contrast to the results from this overproduction strategy, we found that targeting Sec22 with dsRNA in Drosophila cells did not significantly effect L. pneumophila intracellular replication unless combined with disruption of TRAPP components. This difference may be accounted for in two ways. There may be residual Sec22 protein remaining after dsRNA treatment. Alternatively, overproduction of membrin may disrupt multiple SNARE proteins that together decrease L. pneumophila replication. Similar to this second possibility, targeting Sec22 and TRAPP may decrease L. pneumophila intracellular replication by disrupting multiple pathways that promote vesicle docking to the LCV (Figure 7). These pathways act together to promote LCV biogenesis or occur sequentially. The sequential model is particularly attractive for explaining the result of the double knockdown of Arf1 and Sec22, which results in a profound defect in intracellular replication. Sec22 is a SNARE involved in promoting fusion and docking of vesicles that lead in an anterograde fashion from the ER, whereas one activity of Arf1 is regulation of retrograde transport headed toward the ER. Therefore, this double combination may cause the disruption of two separate pathways that normally promote L. pneumophila replication (Figure 7).

Unlike the majority of dsRNA directed against components of the *Drosophila* secretory system, depletion of Cdc48/p97 or several of its binding partners strongly reduced L. pneumophila replication. This protein had not been implicated in promoting replication of intracellular pathogens, and we demonstrated that Cdc48/p97 was also required to support intracellular replication in mammalian cells. This is the first demonstration that the identical host protein is required for intracellular replication in both *Drosophila* and mammalian cells and is a clear demonstration of both the power of the system and the high level of evolutionary conservation of host factors that modulate L. pneumophila intracellular replication.

Cdc48/p97 is a cytosolic chaperone that provides force to dislocate incorrectly folded proteins from the ER [19,21]. One model for Cdc48/p97 function in promoting L. pneumophila replication is that it could provide a cytosolic pulling force on the translocating proteins to help the pushing force of the type IV ATPase. Such a strategy would probably bypass the use of either the Sec61 or Der1 protein channels in the ER, as effector substrates are translocated to the surface of the vacuole through the type IV secretion system, rather using the retrotranslocation channel. The Cdc48/p97 complex may then act as a chaperone to remove these substrates from the type IV channel. There is considerable precedence for Cdc48/p97 acting on membrane-associated proteins in the absence of contribution from the ER-localized channel. For instance, Cdc48/p97 activates a membrane-localized transcription factor in yeast, a function that is separate from retrotranslocation of unfolded proteins [48].

Although Cdc48/p97 is involved in a number of functions in host cells, the analysis of its interacting partners indicates that the important function of this protein in supporting L. pneumophila intracellular replication is to regulate turnover of ubiquitinated substrates. Both Cdc48/p97 and the proteasome control the protein composition of the LCV. Although Cdc48/p97-mediated removal of both ubiquitinated and nonubiquitinated proteins has previously been proposed [18], we cannot eliminate the possibility that the removal of effectors LidA and SidC resulted from low-level ubiquitination of these substrates. Therefore, both the removal of ubiquitin and the removal of effectors may be mechanistically identical processes. Alternatively, as the preponderant fractions of LidA and SidC do not appear to be ubiquitinated (unpublished data), the Cdc48/p97 chaperone may remove translocated effectors without targeting them for destruction, as has been observed for an ER-localized transcription factor in yeast [48]. This may be particularly important for the function of LidA, as removal of this protein from the LCV without subsequent degradation appears to temporally modulate its localization throughout the intracellular replication cycles. Shortly after uptake of L. pneumophila into macrophages, LidA is localized on the LCV, but several hours after replication is established, the protein becomes dispersed throughout the cytoplasm while still maintaining an LCV-associated pool [5]. Thus, there must be factors that modulate the establishment of these two pools of LidA in host cells during L. pneumophila replication.

The final possibility is that the sole function of Cdc48/p97 is to target translocated effectors for destruction. At face value, this would appear to interfere, rather than contribute to intracellular replication. However, little is known about maturation of the LCV, and it is possible that effectors that are important for establishing the LCV would inhibit later maturation. A number of pathogens are able to modulate the host cell ubiquitination system [49,50], but in the case of intravacuolar bacteria this is usually associated with host defense against the pathogen, rather than support of replication and survival within replication vacuoles (Figure 5). Ubiquitination of S. typhimurium that escapes from its replication vacuole results in clearance of the bacterium [17]. In the case of *L. pneumophila,* a positive role for destruction of translocated effectors and other LCV-associated proteins may be envisioned as a mechanism for temporal control of proteins associated with the LCV, similar to that observed for the S. typhimurium SopE protein after uptake of the bacterium into host cells [16]. Little is known about maturation of the LCV during the course of intracellular replication, and we propose that Cdc48/p97 and the ubiquitin proteasome system may monitor the surface of the LCV and change its protein composition as it responds to requirements for intracellular growth that may change over the time period of intracellular residence, thereby promoting high levels of intracellular replication. Although the few translocated effectors that have been localized are associated with the LCV, it is likely that some effectors act elsewhere in the cell. Cdc48/p97 may help complete the translocation process and release these proteins for action. In addition, little is known about host factors that limit intracellular replication, and this class of proteins may be among those targeted for destruction by *L. pneumophila.* Identification of these ubiquitinated proteins will be the first step towards understanding the temporal or spatial control of translocated proteins.

In summary, RNA interference analysis provided crucial evidence for the hypothesis that L. pneumophila exploits functionally redundant host pathways to create its intracellular niche. It is likely that the bacterium injects a large number of effector proteins into the host cell, but how these effector proteins exploit the host pathways identified is unknown. The combination of bacterial genetics and RNA interference should help identify interactions between host and pathogen that lead to the formation and maintenance of the LCV. Ubiquitinated bacterial proteins, controlled by an ERAD-like process, likely define an important subset of translocated effectors that may serve as a switch from establishment of the LCV to an active replicative compartment.

## Materials and Methods

### Cell culture, inhibitors, and RNA silencing.

*Drosophila* Kc167 cell cultures were grown in Schneiders medium plus 10% heat-inactivated fetal bovine serum (GIBCO, San Diego, California, United States). For dsRNA treatment, 1 × 10^6^ cells/ml were plated in Schneiders medium without serum and 5 μg dsRNA was added per 1 × 10^6^ cells for 45 min. One-half volume of Schneiders medium plus serum was added back and cells were incubated for 3–4 d as indicated. dsRNA templates were prepared by PCR from *Drosophila* genomic DNA with the oligos listed in Table S1. PCR products were transcribed using the Megascript RNA kit (Ambion, Austin, Texas, United States). dsRNA quality was assessed by gel electrophoresis. HEK 293 cells were maintained in Dulbecco's Modified Eagle's Medium with 10% heat inactivated fetal bovine serum (Hyclone, South Logan, Utah, United States). GFP-expressing small interfering RNA plasmids [39] created in pRNAT-U6.1/Neo (GenScript, Piscataway, New Jersey, United States) and transfected with lipofectamine 2000 per the manufacturer's protocol. In all experiments, cells were transfected for 4 d with silencing vectors. Mouse BMDMs were prepared as described [8]. In all experiments, the proteasome inhibitor MG-132 (Calbiochem, San Diego, California, United States) was dissolved in DMSO and used at 10 μM; controls were treated with DMSO alone. CM (Sigma, St. Louis, Missouri, United States) was used at 12.5 μg/ml.

### Single and double mutant screen.

For the single mutant screen, *Drosophila* Kc167 cells (1.25 × 10^5^) were plated in 96-well dishes and treated for 3 d with dsRNA. For the double mutant screen, *Drosophila* Kc167 cells (2.5 × 10^4^) were plated in 384-well dishes and treated for 4 d with single or double combinations of dsRNAs. The wells were then incubated with wild-type L. pneumophila at multiplicity of infection (MOI) = 1 for 1 h, washed 2 times, incubated for 30 h, and fixed and immunostained with anti–L. pneumophila. DNA was stained with 1 μg/ml DAPI. Single mutants were screened as described in Table 1. For the double mutant screen, ten master dsRNAs (Sec23, Sec24, Apg6, Sec22, Ykt6, Rab5a, Rab1a, Hrd3, Nsf, and Syn5) were selected to combine with the entire collection. Fluorescein and DAPI images were compared visually to identify double mutant combinations for further study.

### RT-PCR and Western analysis of dsRNA knockdowns**.**

Drosophila cells (1 × 10^6^) were treated with dsRNA against p47 or p97/Cdc48 for 3 d. For analysis of p47, total RNA was isolated with Trizol (Invitrogen, Carlsbad, California, United States) and reverse-transcribed using random primers and Superscript (Invitrogen), and 20 cycles of PCR were performed with primers CCGCAAGAGCTGCTGG and CGACGAATGTCGGAGAC for p47 or primers ATGTGTGACGAAGAAGTTG and AGTCCAGAACGATACCG for Act5c. For analysis of p97/Cdc48 anti-Ter94 (*Drosophila* Cdc48/p97) [35] and antitubulin (Serotec, Raleigh, North Carolina, United States) were used for western blotting. p97/Cdc48 and p47 were silenced in 293 cells as described above. Anti-p47 [20] and anti-p97 (Progen, Queensland, Australia) were used for Western blotting.

### Bacterial Strains and Growth.

L. pneumophila (Philadelphia) strains are derived from serogroup-1; growth was as described [5,8]. S. typhimurium with constitutive GFP (gift of Brad Cookson, Department of Microbiology, University of Washington) were grown at 37 °C in Luria Broth. Y. pseudotuberculosis strain IP2666 was grown to log phase at 23 °C before infection.

### Immunofluorescence, antibodies, and microscopy.

Immunostaining with polyclonal anti–L. pneumophila, anticalnexin (Stressgen Biotechnologies, San Diego, California, United States), monoclonal LAMP-1 antibody IB4D [8], anti-Ter94 (*Drosophila* Cdc48/p97) [35], anti-p47 [20], anti-SidC [4], anti-LidA [5], and antipolyubiquitin FK1 (BIOMOL International/Affiniti, Exeter, United Kingdom) as described. All fixation was in 3.7% formaldehyde for 15 min and permeablization with 0.1% TX-100, unless otherwise noted. Anti-p97 (Progen) was visualized in BMDMs after 15 min of fixation in 3.7% formaldehyde/phosphate-buffered saline and extraction for 10 min at −20 °C in methanol [20]. Quantification of infectious centers and isolation of replication vacuoles were performed as described [5]. For all visual studies, at least 100 cell-associated bacteria were counted, and standard errors were calculated from three coverslips. Linear changes were made to all images, except Figure 3A, in which the background decrease was nonlinear. All microscopy was completed with a 100× objective and images were collected using OpenLAB software (Agilent Technologies/Scientific Software, Pleasanton, California, United States).

## Supporting Information

Figure S1 L. pneumophila Replicates Normally in *Drosophila* Kc167 Cells Kc167 cells were plated in 24-well dishes and incubated with Lp02 (intact type IV translocation system) or Lp03 (*dotA* ^−^ *,* defective for type IV translocation) at MOI = 0.1 for 1 h; the monolayers were then washed and lysed, and bacterial counts were titred for colony-forming units at the indicated times. Each well was plated in duplicate and each timepoint represents triplicate wells. The mean is shown, ± standard error.
(34 KB PDF)

Figure S2 RNAi Directed against Cdc48/p97 and Cofactors Does Not Affect Intracellular Replication of Y. pseudotuberculosis or S. typhimurium
(A) Kc167 cells were treated with dsRNA for 3 d and incubated with Y. pseudotuberculosis for 1 h at MOI = 20. Extracellular bacteria were treated with 100 μg genatmycin for 1 h and washed and incubated for 24 hours. Cells were then fixed and stained with anti*–Y. pseudotuberculosis,* and infectious centers were assayed*.*
(B) Kc167 cells treated as in (A), incubated with S. typhimurium (pGFP) for 1 h at MOI = 5 and washed and incubated for 24 hours with 15 μg/ml gentamycin. Cells were fixed and infectious centers were assayed by fluorescence microscopy.
(C) Kc167 cells were treated with dsRNA as in (A) and incubated with L. pneumophila, MOI = 1, or Y. pseudotuberculosis at MOI = 20 for 1 h then washed and incubated 30 h with L. pneumophila or 24 h with *Y. pseudotuberculosis.* Cells were then fixed, stained with the appropriate antibody, and assayed for infectious centers by fluorescence microscopy.
(54 KB PDF)

Table S1 Oligonucleotide Sequences Used in This Study. (63 KB PDF)

## Abbreviations

BMDM: bone marrow–derived macrophage
CM: chloramphenicol
dsRNA: double-stranded RNA
ER: endoplasmic reticulum
ERAD: ER-associated degradation
GFP: green fluorescent protein
IC: intermediate compartment
LCV: Legionella pneumophila–containing vacuole
MOI: multiplicity of infection
TRAPP: transport protein particle
