# Supplementary figures and images for: RNA Interference Analysis of Legionella in Drosophila Cells: Exploitation of Early Secretory Apparatus Dynamics
Source: PLoS Pathog. 2006 Apr 28;2(4):e34. doi: 10.1371/journal.ppat.0020034 (PMC1447669; doi:10.1371/journal.ppat.0020034)

Supplementary Figure 1

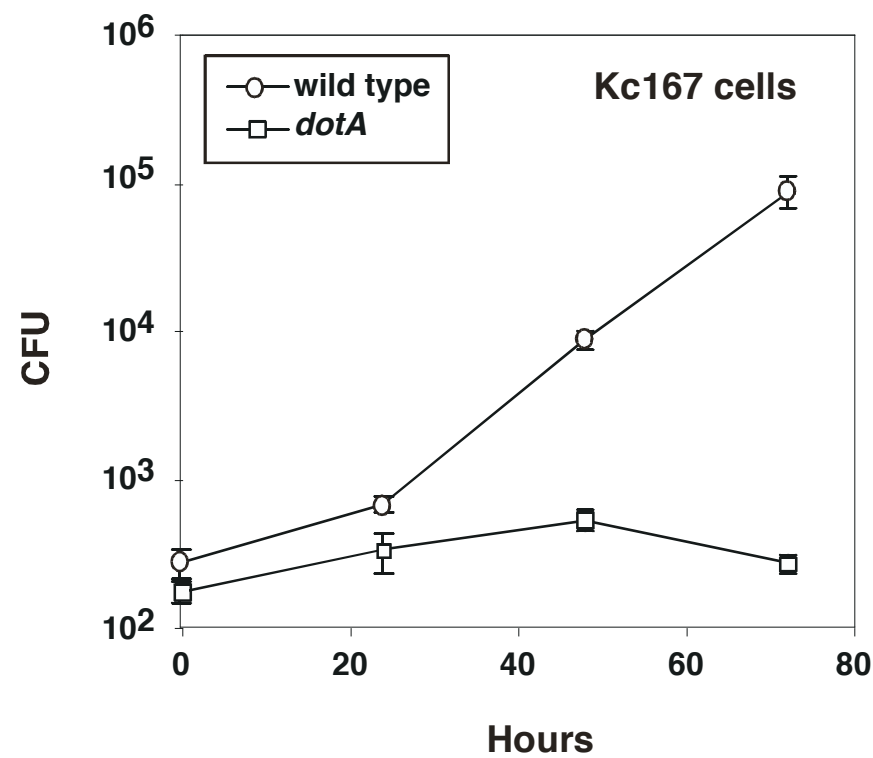

Supplement: Figure S1 — Kc167 cells were plated in 24-well dishes and incubated with Lp02 (intact type IV translocation system) or Lp03 (dotA − , defective for type IV translocation) at MOI = 0.1 for 1 h; the monolayers were then washed and lysed, and bacterial counts were titred for colony-forming units at the indicated times. Each well was plated in duplicate and each timepoint represents triplicate wells. The mean is shown, ± standard error. (34 KB PDF) [file ppat.0020034.sg001.pdf]

Supplementary Figure 2

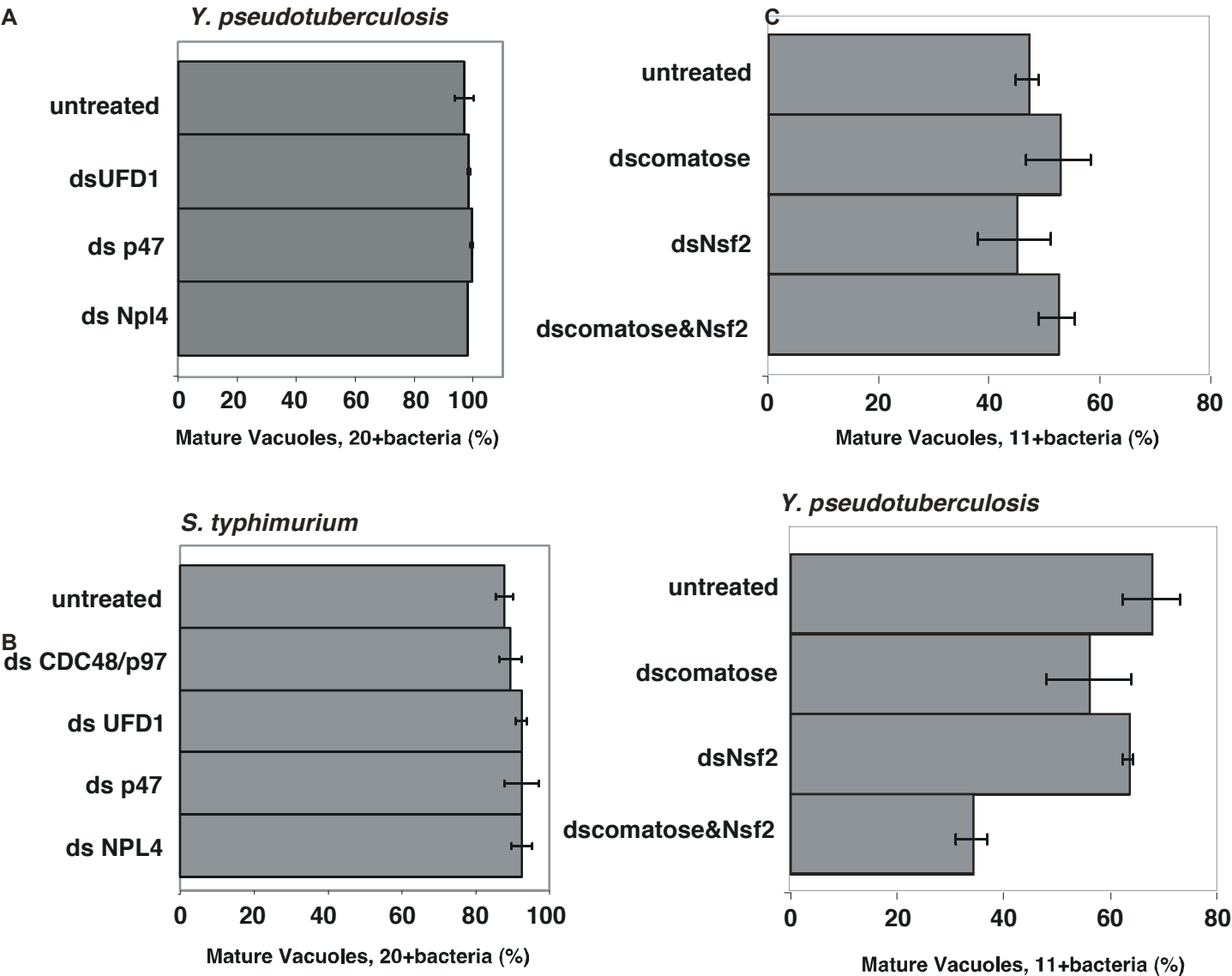

Supplement: Figure S2 — (A) Kc167 cells were treated with dsRNA for 3 d and incubated with Y. pseudotuberculosis for 1 h at MOI = 20. Extracellular bacteria were treated with 100 μg genatmycin for 1 h and washed and incubated for 24 hours. Cells were then fixed and stained with anti–Y. pseudotuberculosis, and infectious centers were assayed. (B) Kc167 cells treated as in (A), incubated with S. typhimurium (pGFP) for 1 h at MOI = 5 and washed and incubated for 24 hours with 15 μg/ml gentamycin. Cells were fixed and infectious centers were assayed by fluorescence microscopy. (C) Kc167 cells were treated with dsRNA as in (A) and incubated with L. pneumophila, MOI = 1, or Y. pseudotuberculosis at MOI = 20 for 1 h then washed and incubated 30 h with L. pneumophila or 24 h with Y. pseudotuberculosis. Cells were then fixed, stained with the appropriate antibody, and assayed for infectious centers by fluorescence microscopy. (54 KB PDF) [file ppat.0020034.sg002.pdf]
