# Supplementary material for: RNA Interference Analysis of Legionella in Drosophila Cells: Exploitation of Early Secretory Apparatus Dynamics
Source: PLoS Pathog. 2006 Apr 28;2(4):e34. doi: 10.1371/journal.ppat.0020034 (PMC1447669; doi:10.1371/journal.ppat.0020034)
Supplement: Table S1 — (63 KB PDF) [file ppat.0020034.st001.pdf]

Supplemental Table 1

Oligonucleotide sequences used in this study.

Gene Name	Accession	Primer 1	Primer2
SEC22	CG7359	gcgcTAATACGACTCACTATAGGGAGACCACgctacctaatcgag	gcgcTAATACGACTCACTATAGGGAGACCACtagaagtatcatgcc
ARF1	CG11027	TAATACGACTCACTATAGGGAGACCACttcaagacgggactgac	TAATACGACTCACTATAGGGAGACCACattggttggacagccag
SEC23	CG1250	TAATACGACTCACTATAGGGAGACCACcctaacatgcaacgtttg	TAATACGACTCACTATAGGGAGACCACcagaagttgcacaccca
SAR1	CG7073	TAATACGACTCACTATAGGGAGACCACTCACACTGGGGCTTATG	TAATACGACTCACTATAGGGAGACCACTGTCAACTGGGCAGCAC
FSG28	CG7700	TAATACGACTCACTATAGGGAGACCACcacaccgattgccacac	TAATACGACTCACTATAGGGAGACCACattcatagtagcacaaagc
fmembrin	CG4780	TAATACGACTCACTATAGGGAGACCACtccaatgttaacgcgtca	TAATACGACTCACTATAGGGAGACCACctgtgattcagcagctg
BET1	G14084	TAATACGACTCACTATAGGGAGACCACtactccgctttggtcac	TAATACGACTCACTATAGGGAGACCACcgtccggtccatgtcg
YKT6	CG1515	TAATACGACTCACTATAGGGAGACCACgaatgccagtgcgctg	TAATACGACTCACTATAGGGAGACCACggcgatcagtacaccg
Arf79F	CG8385	TAATACGACTCACTATAGGGAGACCACctcatgctgacgcattga	TAATACGACTCACTATAGGGAGACCACcggcatccaaaccgac
SYN5	CG4214	TAATACGACTCACTATAGGGAGACCACgtccaacagatcatttcg	TAATACGACTCACTATAGGGAGACCACgttgattgccggtgacc
SLY1	CG3539	TAATACGACTCACTATAGGGAGACCACacgcgttgcctcgcc	TAATACGACTCACTATAGGGAGACCACtgcagcgtcacgccc
SEC1	CG15811	TAATACGACTCACTATAGGGAGACCACgctgatgaacgaggtg	TAATACGACTCACTATAGGGAGACCACccggcgagtagaggc
SEC13	CG6773	TAATACGACTCACTATAGGGAGACCACcacactgctcaaccagg	TAATACGACTCACTATAGGGAGACCACgatacaagacgtccaagc
SEC24	CG1472	TAATACGACTCACTATAGGGAGACCACcctatgaattagcagcatg	TAATACGACTCACTATAGGGAGACCACcggattgttcaacgctg
SEC31	CG8266	TAATACGACTCACTATAGGGAGACCACatgaagatcaaggaactgc	TAATACGACTCACTATAGGGAGACCACcgattcggaggcgcat
ARL1	CG 6025	TAATACGACTCACTATAGGGAGACCACgtagcaccacatacaatta	TAATACGACTCACTATAGGGAGACCACgccgatggtgggtatc
ARL2	CG 7435	TAATACGACTCACTATAGGGAGACCACacaatcctgaagcgctt	TAATACGACTCACTATAGGGAGACCACggatgagagggctccc
ARL4B	CG 2219	TAATACGACTCACTATAGGGAGACCACcattgaagaatgggtgcta	TAATACGACTCACTATAGGGAGACCACtgccatcagtgcaacta
ARF5	CG11027	TAATACGACTCACTATAGGGAGACCACcacagctccaggaggat	TAATACGACTCACTATAGGGAGACCACcagcatacgagaacagc
BET3	CG3911	TAATACGACTCACTATAGGGAGACCACatgctgcgcgacttcg	TAATACGACTCACTATAGGGAGACCACccagctgcaccatctc
BET5	CG1359	TAATACGACTCACTATAGGGAGACCACgcattccataacttcgcc	TAATACGACTCACTATAGGGAGACCACatccagagggagtttcc
TRS23	CG9298	TAATACGACTCACTATAGGGAGACCACtgggccatgtcctggt	TAATACGACTCACTATAGGGAGACCACcgatattgctaatgcccg
VTI1	CG3279	TAATACGACTCACTATAGGGAGACCACgtattactgttcacctc	TAATACGACTCACTATAGGGAGACCACtggcttgggagcgct
SYN6	CG7736	TAATACGACTCACTATAGGGAGACCACagtgttctctgggccat	TAATACGACTCACTATAGGGAGACCACatagtgtcctcgaggtc
SYX8	CG4109	TAATACGACTCACTATAGGGAGACCACgctaccagccatcagc	TAATACGACTCACTATAGGGAGACCACttctggtcctccacctc
SNAP25	CG9474	TAATACGACTCACTATAGGGAGACCACgtcttgcctctcaacca	TAATACGACTCACTATAGGGAGACCACcgtgatcctggccaca
g-SNAP	CG3988	TAATACGACTCACTATAGGGAGACCACcACACTATTTTCAAACG	TAATACGACTCACTATAGGGAGACCACCCAATATGTGTGTATCTTG
SNAP-31	CG6208	TAATACGACTCACTATAGGGAGACCACATGAGCACTCTTGATGCC	TAATACGACTCACTATAGGGAGACCACGAGTCTGACCAATATCCG
SNAP-29	CG11173	TAATACGACTCACTATAGGGAGACCACTGGCCCATAACTACCTG	TAATACGACTCACTATAGGGAGACCACCTTGAGGCCGCCGAAG
ARF 102	CG 31811	TAATACGACTCACTATAGGGAGACCACGAGAACTACAGAACATGG	TAATACGACTCACTATAGGGAGACCACCAGCATACGAGAACAGC
Sec34	CG 3248	TAATACGACTCACTATAGGGAGACCACTGGATGACGTCGAACGA	TAATACGACTCACTATAGGGAGACCACGGCATTCGTCGATCTTG

TER94/CDC48/p97	CG2331	gcgcTAATACGACTCACTATAGGGAGACCACctgctgcctcatc	gcgcTAATACGACTCACTATAGGGAGACCACcagcaaaggatgac
HRD1	CG1937	TAATACGACTCACTATAGGGAGACCACccatctggtagcacgac	TAATACGACTCACTATAGGGAGACCACggcttaggcatgggac
HRD3	CG10221	TAATACGACTCACTATAGGGAGACCACtgagctaccagtaagtca	TAATACGACTCACTATAGGGAGACCACcgtcctggaagcaaact
UFD1	CG6233	TAATACGACTCACTATAGGGAGACCACGCCTCCCTCGGCGC	TAATACGACTCACTATAGGGAGACCACCCATCGAGACGTACG
p47	CG11139	TAATACGACTCACTATAGGGAGACCACCGCAACGCTTAGCGA	TAATACGACTCACTATAGGGAGACCACCGCGCATGACAGTCT
NPL4	CG4673	TAATACGACTCACTATAGGGAGACCACGGTGCCACCACAATG	TAATACGACTCACTATAGGGAGACCACCAGTCCCAGCGCACT
UFD2a	CG 11070	TAATACGACTCACTATAGGGAGACCACGACAAGCATCGAGGAGA	TAATACGACTCACTATAGGGAGACCACGCTCGAACATCTCCATC
UFD2b	CG 9934	TAATACGACTCACTATAGGGAGACCACGATGCGCGCCCGTCG	TAATACGACTCACTATAGGGAGACCACTCCAGGAAGCTGGCAC
DER1	CG14899	TAATACGACTCACTATAGGGAGACCACGCACCTGGACCTGGT	TAATACGACTCACTATAGGGAGACCACGTAGGATAGACGTCC
UFD3	CG5105	TAATACGACTCACTATAGGGAGACCACGCAGGACCACAAGA	TAATACGACTCACTATAGGGAGACCACCGTCATTTCCGCAGG
SEC61	CG9539	TAATACGACTCACTATAGGGAGACCACAGTGCTATGGACTGC	TAATACGACTCACTATAGGGAGACCACGGACGCGGTACCGAG
SRP binding	CG8583	TAATACGACTCACTATAGGGAGACCACCCATCTTGGCCAATG	TAATACGACTCACTATAGGGAGACCACCACCCACTGCCGAGG
DSK2	CG14224	TAATACGACTCACTATAGGGAGACCACGGACAACCTGACGGT	TAATACGACTCACTATAGGGAGACCACCACCGACTCCAGATTC
RAB5a	CG3664	TAATACGACTCACTATAGGGAGACCACattccgcatccacactc	TAATACGACTCACTATAGGGAGACCACcagttgggtgatctatcc
RAB7	CG5915	TAATACGACTCACTATAGGGAGACCACccagtagagatgagagc	TAATACGACTCACTATAGGGAGACCACgcgggtctggaatacc
RAB5c	CG3870	TAATACGACTCACTATAGGGAGACCACactgctgactgcgcgc	TAATACGACTCACTATAGGGAGACCACttacctcgcgagccca
RAB11	CG5771	TAATACGACTCACTATAGGGAGACCACacatctctagtctcgttg	TAATACGACTCACTATAGGGAGACCACatcaaccttccgcgatc
RAB4a	CG5915	TAATACGACTCACTATAGGGAGACCACacagctgttgtcgcact	TAATACGACTCACTATAGGGAGACCACcggcgaacttgtagtac
RAB4c	CG 4921	TAATACGACTCACTATAGGGAGACCACacagaacttcaagggcag	TAATACGACTCACTATAGGGAGACCACtcggccagacttcagg
RAB1a	CG3320	TAATACGACTCACTATAGGGAGACCACcagtcgccattgttctc	TAATACGACTCACTATAGGGAGACCACgtggggctgctcgataa
SYN13	CG11278	TAATACGACTCACTATAGGGAGACCACcaacgatgcgctgcac	TAATACGACTCACTATAGGGAGACCACctcccgcagatttggc
VPS33a	CG12230	TAATACGACTCACTATAGGGAGACCACgccgaaatccgtgggc	TAATACGACTCACTATAGGGAGACCACggccagctgctccatc
VPS33b	CG5127	TAATACGACTCACTATAGGGAGACCACCTTTACGAGTGTCTCTAC	TAATACGACTCACTATAGGGAGACCACGCCTAAGGACTTTGCTC
VPS45	CG8228	TAATACGACTCACTATAGGGAGACCACCAGCAACATCATTCCCC	TAATACGACTCACTATAGGGAGACCACGTGGTCGTTCGCCTTG
HRS	CG2903	TAATACGACTCACTATAGGGAGACCACCCAGCTCGCAAGACCG	TAATACGACTCACTATAGGGAGACCACCGAGGTCAGGGTCATG
EEA1	CG8506	TAATACGACTCACTATAGGGAGACCACCGACAGCGACGAAGATC	TAATACGACTCACTATAGGGAGACCACGCCTCCGCACAAACGG
coronin	CG4532	TAATACGACTCACTATAGGGAGACCACCGGAGCAGCGGAGCG	TAATACGACTCACTATAGGGAGACCACAGGCAATCCTGCGAGG
flotillin	CG8200	TAATACGACTCACTATAGGGAGACCACAGAATTTCGTTGAACACG	TAATACGACTCACTATAGGGAGACCACCTCATCGCGCAAATCCT
NSF/comt	CG 1618	TAATACGACTCACTATAGGGAGACCACGGATATAGTTCACAATTC	TAATACGACTCACTATAGGGAGACCACGCCCGATTGGTCAGCG
NSF2	CG9931	TAATACGACTCACTATAGGGAGACCACCACAGCATGGCGTGA	TAATACGACTCACTATAGGGAGACCACCTGGGCCAGATTAGCG
TSC1	CG6147	TAATACGACTCACTATAGGGAGACCACCCGCTGATGAGCGCC	TAATACGACTCACTATAGGGAGACCACCATAGCTGCCGTCTT
Rabaptin5	CG4030	TAATACGACTCACTATAGGGAGACCACGGCTCCTGAGGAGGC	TAATACGACTCACTATAGGGAGACCACGCGCTGGATCTGCTT
TRAM	CG11642	TAATACGACTCACTATAGGGAGACCACCCGGGACTCGGTCG	TAATACGACTCACTATAGGGAGACCACGCCCTCCCACAGCTG
cofilin	CG6873	TAATACGACTCACTATAGGGAGACCACCCGGGACTCGGTCG	TAATACGACTCACTATAGGGAGACCACGCCCTCCCACAGCTG
Unknown	CG16251	TAATACGACTCACTATAGGGAGACCACGTATCGTATTTCGAGATC	TAATACGACTCACTATAGGGAGACCACAGACCTCATTGACTGCC
Totc	CG31508	TAATACGACTCACTATAGGGAGACCACATGCCTTGCCCTGCTC	TAATACGACTCACTATAGGGAGACCACTCCAGTAAAGAGCCTGG
Unknown	CG1227	TAATACGACTCACTATAGGGAGACCACGGGGCTTCAGTCTAATC	TAATACGACTCACTATAGGGAGACCACGGGCTTCTGTCATTGAC
RAD23	CG1836	TAATACGACTCACTATAGGGAGACCACCGAGGAGCGCGGTC	TAATACGACTCACTATAGGGAGACCACGCAGCCATCGCACGTT
RPN11	CG18174	TAATACGACTCACTATAGGGAGACCACCTGCTACGTCTTGGAGG	TAATACGACTCACTATAGGGAGACCACCGGACAACGTGAGGC
DOM3Z	CG32566	TAATACGACTCACTATAGGGAGACCACTGGCAACCAAGGACA	TAATACGACTCACTATAGGGAGACCACGCATTATCTCGACGTTAAC

UBA2	CG7528	TAATACGACTCACTATAGGGAGACCACCTCCTGATGCTGCTG	TAATACGACTCACTATAGGGAGACCACCCATGGCTGGCTGGT
PAC10	CG6719	TAATACGACTCACTATAGGGAGACCACGGAGGAGATCGACA	TAATACGACTCACTATAGGGAGACCACTCCTGATCGTGCTCC
PGRP-LA	CG32042	TAATACGACTCACTATAGGGAGACCACACTGGCGCGCCAGCT	TAATACGACTCACTATAGGGAGACCACTGCAGCCGTTTGCATGG
PGRP-LE	CG8995	TAATACGACTCACTATAGGGAGACCACCACCACGGCACCTGC	TAATACGACTCACTATAGGGAGACCACGAGTGCCTAGTGTCATAG
PGRP-lF	CG4437	TAATACGACTCACTATAGGGAGACCACAGTTGGCATCGGACGTT	TAATACGACTCACTATAGGGAGACCACCGCATCCTTCGGTTGC
DOA10	CG1317	TAATACGACTCACTATAGGGAGACCACGGACGACCTGTCGCAG	TAATACGACTCACTATAGGGAGACCACCTCGCGCAGCCAGAC
CHIP	CG5203	AATACGACTCACTATAGGGAGACCACCCCGTTGAAGGTGCTT	TAATACGACTCACTATAGGGAGACCACTGACGACCAAGCACATC
Parkin	CG10523	TAATACGACTCACTATAGGGAGACCACCCCCTGCGCATCGC	TAATACGACTCACTATAGGGAGACCACGGGTCCACGGTGTACT
